# Adherence to guideline recommendations for asthma care in community pharmacies: actual and needed performance

**DOI:** 10.1038/s41533-019-0139-5

**Published:** 2019-07-11

**Authors:** Esther Kuipers, Michel Wensing, Elaine Wong-Go, Bernard J. G. Daemen, Peter A. G. M. De Smet, Martina Teichert

**Affiliations:** 1 Department of IQ Healthcare, Radboud Institute for Health Sciences, Radboud University Medical Centre, PO Box 9101, 6500 HB Nijmegen, The Netherlands; 2 BENU Apotheek Zeist West, Zeist, The Netherlands; 3 Department of General Practice and Health Services Research, University Hospital Heidelberg, Heidelberg, Germany; 4 Royal Dutch Association for the Advancement of Pharmacy (KNMP), Guideline Development, The Hague, The Netherlands; 5 Department of Clinical Pharmacy, Radboud Institute for Health Sciences, Radboud University Medical Centre, Nijmegen, The Netherlands; 6 Department of Clinical Pharmacy & Toxicology, Leiden University Medical Centre, Leiden, The Netherlands

**Keywords:** Health services, Respiratory tract diseases

## Abstract

Pharmaceutical care guidelines aim to provide recommendations for pharmaceutical care, reduce unwanted pharmacy practice variation and ultimately improve the quality of healthcare. This study evaluated community pharmacists’ adherence to recommendations for the provision of care to asthma patients with first dispensing and follow-up refill encounters in The Netherlands. Data were pharmacists’ self-assessment of adherence to guideline recommendations, independent observations of dispensing encounters and a nationwide questionnaire on pharmacists’ views on the desirable (clinical) necessity of applying guideline recommendations to their patient population. The 21 pharmacists who performed self-assessment judged their adherence concerning inhalation instructions as high. The lowest scores were reported for recommendations to collect additional information on the type of lung disease and for asking patients’ expectations, wishes and concerns. Sixty-eight dispensing encounters were observed. In 83% of the 35 first dispensing observations, inhalation instruction was provided. This percentage was lower (62%) at refill dispensings. During all encounters, pharmacy staff seldom explored patients’ perceptions or responded to patients’ expectations, wishes and concerns. One hundred and four pharmacists completed the feasibility questionnaire. Pharmacists judged that all patients should receive inhalation instruction at first dispensing. They regarded it necessary to check on patients’ expectations, wishes and concerns regarding the treatment for only up to 70% of the patients. More efforts on guideline implementation are needed, especially on follow-up dispensings and on gaining relevant information from patients and other healthcare professionals. Pharmacists still have opportunities to grow in applying a patient-tailored approach and exploring patients’ individual needs, rather than providing practical information.

## Introduction

Pharmaceutical care guidelines aim to describe optimal pharmaceutical care (i.e. timely and appropriate patient-centred care, tailored to the individual patient’s needs), the care patients and stakeholders can expect from pharmacists and ultimately improve the quality of healthcare by providing pharmacists with recommendations that reflect prevailing knowledge.^1,2^ The Royal Dutch Association for the Advancement of Pharmacy (KNMP) has recently developed a new guideline for asthma care.^3^ The guideline recommendations address pharmacotherapy in asthma, multidisciplinary cooperation with other healthcare professionals (e.g. general practitioners (GPs) and lung specialists), the dispensing process, patient monitoring and counselling. By providing pharmaceutical care to patients with asthma, the pharmacist can help them to achieve treatment goals, e.g. improvement of disease control and reduction of asthma symptoms, exacerbations and medication-related side effects.^4–10^ The pharmacists and their team have an important role in medication counselling, especially at the encounters during the dispensing moments in daily practice. They have good opportunities to inform and counsel patients and support them in using their medication properly.^11–14^ After treatment initiation with a pharmaceutical encounter during the first dispensing (FD) of asthma maintenance medication, patients return to the pharmacy for the first refill (second dispensing (SD)), followed by general refill dispensings (RDs). The guideline contains 23 recommendations for pharmaceutical encounters (14 for FDs, 7 for SDs and 2 for RDs) and emphasises the importance of patient counselling. Especially, information on using the inhaler correctly is essential, because incorrect inhaler technique and non-adherence to therapy are recognised as major factors in poorly controlled or uncontrolled asthma.^15–17^ The information during the FD is targeted to the starting patient, focussing on the inhalation instruction, tailored information to encourage good drug use and the appropriateness of the inhaler for the individual patient. The guideline recommendations regarding SDs focus on patients’ first experiences with the medication (e.g. inhalation technique, effect, side effects).

The period between the first and the second prescription is decisive for the start and subsequent adherence to the medication scheme.^11–14^ During the consultation of a healthcare professional, there are two needs that have to be met: ‘the need to understand’ (to know and understand what is the matter) and ‘the need to be understood’ (to know that he/she has taken seriously and accepted).^18^ To meet both pharmacists’ and patients’ needs, the pharmacist should alternate information-giving and information-asking during patient encounters.^18^ Regarding pharmaceutical encounters in general, the guideline states that these are ideally based on reciprocal trust and shared decision-making, according to the Calgary–Cambridge model.^19,20^ Good communicative skills of pharmacists can encourage active patient participation, which is likely to be associated with positive health outcomes.^21–23^

However, an effect on clinical practice only can be attained by successful implementation of the guideline recommendations into daily routines.^24–26^ There are several success factors and barriers that may enhance or impede the implementation of the recommendations in daily practice.^1,24–30^ The guideline development follows a standardised process and consecutively involves drafting the text based on the existing literature and expertise, early assessment in daily practice, asking experts and organisations for feedback and accreditation and publication. Therefore, before guideline authorisation, a practice test on the actual situation and assessment of the feasibility for implementation has to be performed. In previous studies, adherence to different asthma care guidelines was mainly assessed in surveys by self-assessment of the healthcare professionals^29,31^ or by retrospective extraction from patient records.^32,33^ However, it is obvious that not all care-related activities are documented completely and uniformly, and studies have shown that observation in daily practice also can provide meaningful additional information.^34,35^ Furthermore, it may not always be necessary or even wanted to apply the guideline recommendations to all patients individually. Thus the expectation of finding complete follow-up of all guideline recommendations for the patient population cannot be met from a patient-centred approach. The estimation of healthcare providers on the scores for their population can help to achieve a realistic perspective on the feasibility of adherence to guideline recommendations in clinical practice.

In this study, we aimed to assess the actual performance of community pharmacists regarding recommendations for different pharmaceutical encounters from the concept asthma care guideline, using (1) pharmacists’ self-assessment, (2) real-time observations and (3) a questionnaire on the scores to be achieved on population level for guideline recommendations with regard to individual patient needs.

## Results

### Basic pharmacy characteristics

All pharmacists from 21 community pharmacies reported that they worked according to a certified quality management system. Overall, they reported that they cooperated well with GPs in structured pharmacotherapy audit meetings. A consulting room was available in all pharmacies. The mean team size was 1.71 (SD 0.83, range 0.85–3.4) fulltime equivalent (FTE) pharmacists and 8.11 (SD 3.27, range 4–15) FTE pharmacy assistants.

### Self-assessment

Twenty-one pharmacists completed the self-reported adherence questionnaire on the implementation of the guideline recommendations regarding the three types of dispensing encounters. Regarding the FD encounters, for 5 out of 14 items, the majority (≥16 of the 21 pharmacists) reported to be 80–100% adherent (Table 1). These included checking the appropriateness of the inhaler for the patient, checking if inhalation instruction already had been provided, giving inhalation instruction if necessary, using the protocols of the Lung Alliance Netherlands (LAN) for the instruction and making sure that all information was understood by the patient. Nine recommendations showed lower adherence rates, with the lowest scores for verification of the type of lung disease; checking patients’ expectations, wishes and concerns regarding the treatment; and making appointments for follow-up consultation or repeated inhalation instruction.

**Table 1:** Adherence to guideline recommendations from pharmacists’ self-report, independent observations and reported necessity for adherence

	Self-reported adherence (frequency)^a^ We do this in ....% of the situations				Observed adherence^b^ [% of the encounters]	Necessary adherence^c^ for patient population [%], (IQR)^d^
	0–20%	20–50%	50–80%	80–100%		
*Recommendations for first dispensing*						
Check whether the inhaler is appropriate for the patient	1	1	3	16	68.6	90 (72.5–100)
Use the LAN protocols for inhalation instruction	—	—	5	16	91.4	90 (80–100)
Check if the patient has already received inhalation instruction from another healthcare provider	—	—	4	17	68.6	90 (80–100)
Give inhalation instruction (or make sure that another healthcare provider did, dependent on local agreements)	1	—	1	19	82.9	100 (90–100)
Verify the type of lung disease with the patient	10	6	3	2	48.6	80 (60–90)
Check patients’ expectations, wishes and concerns regarding the treatment	8	6	4	3	22.9	70 (50–80)
Check what the patient already knows about asthma and the treatment	4	7	6	4	40.0	70 (60–80)
Check what the patient already knows about the prescribed medicine	—	5	6	10	68.6	80 (70–90)
Provide tailored advice focussed on patients’ individual needs	1	3	6	11	62.9	80 (70–97.5)
Agree with the patient on subsequent counselling with inhalation instruction	11	5	3	2	2.9	70 (50–87.5)
Check whether the patient understood the information	—	1	3	17	97.1	90 (80–100)
Address service possibilities in the pharmacy (e.g. repeated prescription service, e-health, etc.)	1	5	7	8	28.6	70 (50–80)
Make an appointment for follow-up consultation in the pharmacy	13	5	2	1	2.9	70 (40–80)
Note all relevant findings and follow-up appointments (if appropriate)	5	3	5	8	14.3	80 (60–100)
*Recommendations for second dispensing*						
Discuss agreements from the first dispensing encounter (if appropriate)	8	9	2	2	46.2	70 (52.5–90)
Ask patient’s experiences with the medication and possible problems	2	5	5	9	100.0	80 (70–97.5)
Check patients’ expectations, wishes and concerns regarding the treatment	10	6	4	1	15.4	70 (60–80)
Check the inhalation technique and repeat the instruction if necessary	2	8	5	6	61.5	80 (60–100)
Check whether it is needed to discuss topics that were unclear during the first dispensing or in need of repetition	6	8	5	2	30.8	70 (52.5–80)
Check whether the patient understood the information	—	1	8	12	84.6	90 (70–100)
Pay attention to the possibilities for follow-up consultation	9	4	4	4	15.4	70 (50–80)
*Recommendations for follow-up dispensing*						
Ask patient’s experiences with the medication and possible problems	9	8	2	2	40.0	70 (60–90)
Actively screen for suboptimal use of inhalation medication (e.g. overuse of SABA or medication non-adherence)	4	5	3	9	25.0	80 (60–90)

*LAN* Lung Alliance Netherlands, *SABA* short-acting beta-agonist

^a^*n* = 21

^b^*n* = 35 for FD, *n* = 13 for SD, *n* = 20 for RD

^c^*n* = 104

^d^IQR: interquartile range (quartile 1–quartile 3)

During the SD encounters, the highest score was found for checking whether the patient had understood the information. The lowest scores were reported for checking patients’ expectations, wishes and concerns regarding the treatment and discussing agreements from the FD encounter (10 and 8 pharmacists do this in 0% to 20% of the situations, respectively).

Regarding the RD encounters, 17 out of the 21 pharmacists reported that they check patients’ medication experiences in <50% of the situations.

### Real-time observations

Sixty-eight individual pharmaceutical encounters were observed: 35 FD, 13 SD, and 20 RD encounters (Table 1). For 3 out of the 14 items regarding the 35 FD encounters, the observed adherence rate was at least 80%. This included providing inhalation instruction (83%), using the LAN protocols for the instruction (91%) and, at the end of the counselling, making sure that all information was understood by the patient (97%). For 4 items, the adherence rates were between 60% and 70%, including checking the appropriateness of the inhaler, assessing whether the patient had already received inhalation instruction from another healthcare provider, checking what was known about the prescribed medication and providing tailored advice. The lowest adherence rates (3%) were found for the recommendations on appointments for a SD consultation for repeating the inhalation instruction. Checking patients’ expectations, needs and concerns regarding the treatment (23%) and documenting all relevant findings and follow-up appointments (14%) were also not common during the FD observations. During the SD encounters, adherence rates remained low (15%) with respect to these two recommendations. Patients’ experiences and possible problems were always (100%) discussed in SD encounters and in 40% of the RD encounters.

During the RD encounters, active screening on suboptimal use of inhalation medication (e.g. overuse of short-acting beta-agonist or maintenance medication non-adherence) was observed in 25% of the follow-up refill encounters.

### Necessity questionnaire for adherence to guideline recommendations at the population level

One hundred and four expert pharmacists (response rate 5.4%) completed the questionnaire regarding the expected necessity of adherence to the 23 recommendations at the population level regarding the needs of individual patients in daily practice (Table 1).

The highest scores (adherence up to 90%) were reported on the recommendations regarding checking the appropriateness of the inhaler, using the LAN protocols, giving inhalation instruction and checking whether the patient understood the information.

The highest rate of consensus was for providing inhalation instruction. The lowest rate of consensus was for the FD recommendations regarding appointments for follow-up consultations (median score 70%, interquartile range (IQR) 40–80) and documenting the relevant findings (median score 80% IQR 60–100) and for the SD encounters for checking the inhalation technique (median score 80%, IQR 60–100) and discussing agreements from the FD encounter (median score 70%, IQR 52.5–90).

Overall, for 3 out of the 23 recommendations, the performance observed in daily practice reached the desired score. First, using the LAN protocols was observed in 91.4% of the FD encounters (necessity score of 90%). Second, checking whether the patient has understood the information was reported to be necessary for at least 90% of the patients, and this was observed in 97.1% of the FD encounters. Patient’s experiences with the medication and possible problems were asked in 100% of the SD encounters (necessity score of 80%). However, the pharmacists seemed to have underestimated their performance; the self-reported adherence was lower for these recommendations.

The largest differences in observed adherence and desired scores were found for the following recommendations: making an appointment for follow-up consultation in the pharmacy (2.9% observed vs. 70% desired); agreeing with the patient on subsequent counselling with inhalation instruction (2.9% vs. 70%); noting all relevant findings and follow-up appointments (14.3% vs. 80%); checking patients’ expectations, wishes and concerns regarding the treatment (15.4% vs. 70%); and paying attention to the possibilities for follow-up consultation (15.4% vs. 70%).

## Discussion

This study provided insights into the actual situation and assessment of the feasibility of implementing recommendations from the concept asthma care guideline for community pharmacies in daily practice.

Overall, adherence to the guidelines varied across recommendations as well as pharmacists. There were similarities but also differences between the self-reported adherence, real-time observations and desired scores. This study showed that pharmacy staff members were focussed on providing relevant information during the FD encounters, but the information was less comprehensive during RDs. First refills were not always recognised and acted upon, and when there were no appointments made after the FD, patients were possibly not aware of the opportunity for additional care activities.

To fulfil pharmacists’ and patients' needs, the roles of information-giving and information-seeking have to be alternated during encounters. However, pharmacy assistants rarely explored patients’ perceptions or asked about expectations, wishes and concerns. This has also been shown in earlier studies; pharmacy staff in community pharmacies generally provided practical information and seldom discussed patient’s preferences and perceptions about prescribed medications.^11,14^ Specifically, the topics for repeated dispensings were rarely discussed and the pharmacy staff members did not stimulate patients to ask questions, which is also found in other studies.^14,21,36–39^ In addition, the observers mentioned that, although in the majority of the encounters pharmacists checked whether the patient had understood the information, this was asked in a rhetorical way rather than with an open question.

The results of the self-assessment and observations were added to by our questionnaire survey. In general, there was a lack of consensus among pharmacists. Clearly, there are some recommendations that should be followed for the large majority of the patients, for example, providing inhalation instruction or checking whether patients had understood all information and the appropriateness of the prescribed inhaler. However, pharmacists reported lower feasibility scores for the majority of recommendations. Some of these recommendations (e.g. making appointments for follow-up consultations or addressing service possibilities) could be considered as optional, dependent on patients’ needs.

To improve patient outcomes, it is important to apply such a patient-tailored approach and adapt the provided care to the needs of the individual patient.^15,40,41^ Earlier studies have shown that patients with chronic conditions have a need and a desire for pharmacist counselling about new medications and that providing patients with appropriate, tailored information has the potential to improve medication adherence.^42–44^ However, to apply a patient-tailored approach, it should be even more important to recognise those needs in daily practice.

In this study, pharmacists reported in the questionnaire that checking patients’ expectations, wishes and concerns regarding the treatment was desirable in just 70% of the patients, but during the observations, the score was much lower (22.9% for FD, 15.4% for SD). Apparently, pharmacists are still used to a protocol-driven way of providing information. However, to extend the traditional role of the pharmacist towards the provision of patient-centred pharmaceutical care, pharmacists should focus more on discussing patient’s preferences, perceptions and needs rather than providing practical information. After all, it is difficult to provide patient-centred care when the type of lung disease (and consequently the guideline that should be applied) is not known or when patients’ needs are not explored.

This study was a small-scale assessment of adherence to guidelines in daily practice, combining observed practice and reported intention regarding the adherence of guideline recommendations. The independent observers considered the implementation of the guideline from a theoretical perspective, and the trained and educated pharmacists were experienced in working according to the guidelines in daily practice. This study was a first step towards evaluating guideline adherence and facilitating further research that explores barriers for poor adherence.

There are some limitations to our study. First, the self-assessment and observations were conducted in a small, convenience sample of 21 pharmacies; however, their characteristics did not differ from the national data.^45^ Second, the real-time observations were performed at single moments (‘snapshots’), which may have led to bias. However, all pharmacists were experienced in pharmacy practice research and education and were open to critical self-reflection. Third, the pharmacists were aware that they were being observed and thus the performances noted may have been better than normal performances. Consequently, the potential for improvement in clinical practice may be even higher for pharmacies in The Netherlands. Finally, because the feasibility questionnaire was not validated beforehand, some pharmacists might have experienced difficulty in comprehending the instruction or the language used.

The guideline recommendations are formulated for the best quality of care to be provided from actual knowledge. With subsequent implementation of these recommendations, it is expected that pharmaceutical care will be improved. In conclusion, this study showed that, for a select group of leading pharmacists, performance on information supply was high and performance in exploring individual patient’s needs was low. More efforts on guideline implementation are needed, especially on follow-up dispensings and on gaining relevant information from patients and other healthcare professionals. These results indicate that more effort is needed to change the role of the pharmacist from a professional who dispenses practical information to that of a patient-tailored coach during medication use, according to the guideline recommendations. More research is needed to explore the barriers in clinical practice to change the behaviour of pharmacists and technicians.

## Methods

### Ethical approval

The study protocol was approved by the Ethical Committee of the Radboud UMC Nijmegen (approval number: 2018–5057). We have complied with all relevant ethical regulations.

### Design

This observational study consisted of several elements: (1) pharmacists’ self-assessment of adherence to guidelines, (2) real-time observations of adherence to guidelines in daily practice, and (3) a nationwide questionnaire on pharmacists’ assessment of the necessity to follow the recommendations at a population level.

### Setting

A convenience sample of 21 community pharmacies in The Netherlands was available for the self-assessment and real-time observations. During their second year of master’s education in the University of Leiden, 21 pharmacy students followed their internship in community pharmacies, under the supervision of practising pharmacists who were specialised in community pharmacy and were trained to supervise students. Before the start of their internship, the students were educated in asthma symptoms (e.g. shortness of breath, wheezing sound when exhaling), treatment and pharmaceutical care according to the professional guideline. Additionally, they received a briefing and written instructions about their role of observers in the research project. All pharmacists verbally agreed to participate in this study as part of a practical assignment during the internships.

Pharmaceutical care in community pharmacies in The Netherlands has been previously described in detail.^13,46^ Because most patients in The Netherlands attend a single community pharmacy, pharmacists usually possess the complete medication histories of their patients.^47,48^ Community pharmacists and GPs had regular structured pharmacotherapy audit meetings since 1990. In these local meetings, they make agreements on pharmacotherapy based on national guidelines to improve prescribing and dispensing of drugs.^49,50^

### Guideline recommendations

The researchers selected the 23 guideline recommendations for pharmaceutical encounters (14 for FDs, 7 for SDs and 2 for RDs) from the concept asthma guideline. Dispensings are defined as FD when there was no dispensing of the same medication in the year before. All further following dispensing encounters after SD were defined as RDs.

During the RDs, the guideline recommends discussing patients’ experiences and possible problems, if necessary. After patients had been using the medications for a period of time, the pharmacist was advised to screen the patients on drug therapy-related problems and suboptimal medication use (e.g. overuse of rescue mediation, underuse of maintenance medication, inappropriate inhaler use or non-adherence) during the encounters or by clinical decision support systems.^51^

### Measures

For each of the 23 recommendations, the pharmacists answered the self-assessment questionnaire on a Likert scale with the options: ‘we do this in 0–20% of the situations’, ‘we do this in 20–50% of the situations’, ‘we do this in 50–80% of the situations’, and ‘we do this in 80–100% of the situations’. Pharmacy characteristics (e.g. team size and the availability of a consulting room) were collected.

Each student performed real-time observations of pharmaceutical encounters by the pharmacy staff (both pharmacists and pharmacy assistants) in daily practice during the internship. They noted their observations using a standardised scoring list with ‘yes’ and ‘no’ options for each of the 23 selected recommendations. In addition, they were invited to reflect freely on their personal observations, answering the question ‘what did you notice in this encounter?’ All students were educated on asthma symptoms, the medications and the guideline recommendations. They received additional training on the use of the checklists and instructions to observe at least two dispensing encounters in daily practice. They were independent observers and did not intervene.

All pharmacists in The Netherlands were invited to complete a questionnaire regarding the necessity to follow the 23 selected recommendations at the population level. They were asked to score the minimum level (%) of adherence for each recommendation. To determine this, pharmacists were asked to consider the need to apply certain recommendations (e.g. do all patients need inhalation instruction, do all patients need a repeated instruction) only as they pertain to individual patients and to assume that all organisational preconditions (e.g. time, incentives, skills of the pharmacy staff, computer support) were met. They answered questions ‘for which minimum percentage of patients should this recommendation be ideally followed for optimal implementation of this guideline recommendation in clinical practice?’ on a 10-point Likert scale with 10 categories: 0–10% of the patients, 11%–20%, 21%–30%, etc. An e-mail invitation to participate in the survey was sent to 1936 community pharmacies in The Netherlands. Non-responders were sent a reminder 1 week later. The questionnaire was distributed nationwide to give all pharmacists the opportunity to share their opinions, but pharmacists from the special interest group on lung diseases from the KNMP and the pharmacy practice network were specifically invited to complete the questionnaire. Together with the questionnaire, the respondents received an instruction to focus on the desirable (clinical) necessity for the patients and not on the practical (organisational) feasibility in the pharmacy.

### Data analysis

Data from the self-assessment and observations were documented in Microsoft Word 2010. Descriptive statistics were used. For the necessity questionnaire, we reported the upper value of the category as scored by the pharmacists. Depending on the type of variable, the measures of dispersion were analysed using median and IQR for non-normally distributed variables and mean and SD for the normally distributed variables. All analyses were performed using IBM Corp SPSS statistics, Chicago IL, USA, version 25.

### Reporting summary

Further information on experimental design is available in the Nature Research Reporting Summary linked to this article.

## Supplementary Information

Reporting Summary

## Data Availability

The authors declare that all data supporting the findings of this study are available within the paper.

## Supplementary Information

**Supplementary Information** accompanies the paper on the *npj Primary Care Respiratory Medicine* website (10.1038/s41533-019-0139-5)

## References

[1] Woolf SH, Grol R, Hutchinson A, Eccles M, Grimshaw J (1999). Clinical guidelines: potential benefits, limitations, and harms of clinical guidelines. BMJ.

[2] Hakkennes S, Dodd K (2008). Guideline implementation in allied health professions: a systematic review of the literature. Qual. Saf. Health Care.

[3] KNMP. KNMP-procedure development guidelines for the community pharmacist https://www.knmp.nl/downloads/procedure-knmp-richtlijnen.pdf/at_download/file (2018).

[4] *Global Strategy for Asthma Management and Prevention, Global Initiative for Asthma (GINA)* http://www.ginasthma.org. Archived by WebCite® at http://www.webcitation.org/75oQTDICX

[5] Burke Hannah, Davis Jenny, Evans Sian, Flower Laura, Tan Andrew, Kurukulaaratchy Ramesh J. (2016). A multidisciplinary team case management approach reduces the burden of frequent asthma admissions. ERJ Open Research.

[6] Pike KC, Levy ML, Moreiras J, Fleming L (2018). Managing problematic severe asthma: beyond the guidelines. Arch. Dis. Child..

[7] Garcia-Cardenas V (2016). Pharmacists' interventions on clinical asthma outcomes: a systematic review. Eur. Respir. J..

[8] Garcia-Cardenas V (2013). Effect of a pharmacist intervention on asthma control. A cluster randomised trial. Respir. Med..

[9] Kuipers E, Wensing M, de Smet P, Teichert M (2017). Self-management research of asthma and good drug use (SMARAGD study): a pilot trial. Int. J. Clin. Pharm..

[10] Stuurman-Bieze AG, van den Berg PB, Tromp TF, de Jong-van den Berg LT (2004). Computer-assisted medication review for asthmatic patients as a basis for intervention. Constructing and validating an algorithmic computer instrument in pharmacy practice. Pharm. World Sci..

[11] Koster ES (2015). Patient-provider interaction during medication encounters: a study in outpatient pharmacies in the Netherlands. Patient Educ. Couns..

[12] Ax F, Branstad JO, Westerlund T (2010). Pharmacy counselling models: a means to improve drug use. J. Clin. Pharm. Ther..

[13] van Mil JW (2005). Pharmaceutical care in community pharmacy: practice and research in the Netherlands. Ann. Pharmacother..

[14] van Dijk M (2016). Patient-provider communication about medication use at the community pharmacy counter. Int. J. Pharm. Pract..

[15] Armour CL (2011). Using the community pharmacy to identify patients at risk of poor asthma control and factors which contribute to this poor control. J. Asthma.

[16] Basheti IA, Reddel HK, Armour CL, Bosnic-Anticevich SZ (2007). Improved asthma outcomes with a simple inhaler technique intervention by community pharmacists. J. Allergy Clin. Immunol..

[17] Lavorini F (2013). Switching from branded to generic inhaled medications: potential impact on asthma and COPD. Expert Opin. Drug Deliv..

[18] Ong LM, de Haes JC, Hoos AM, Lammes FB (1995). Doctor-patient communication: a review of the literature. Soc. Sci. Med. (1982).

[19] Kurtz SM, Silverman JD (1996). The Calgary-Cambridge Referenced Observation Guides: an aid to defining the curriculum and organizing the teaching in communication training programmes. Med. Educ..

[20] Greenhill N, Anderson C, Avery A, Pilnick A (2011). Analysis of pharmacist-patient communication using the Calgary-Cambridge guide. Patient Educ. Couns..

[21] Stevenson FA, Cox K, Britten N, Dundar Y (2004). A systematic review of the research on communication between patients and health care professionals about medicines: the consequences for concordance. Health Expect..

[22] Barry CA, Stevenson FA, Britten N, Barber N, Bradley CP (2001). Giving voice to the lifeworld. More humane, more effective medical care? A qualitative study of doctor-patient communication in general practice. Soc. Sci. Med. (1982).

[23] Bodenheimer T, Lorig K, Holman H, Grumbach K (2002). Patient self-management of chronic disease in primary care. JAMA.

[24] Grimshaw JM, Russell IT (1993). Effect of clinical guidelines on medical practice: a systematic review of rigorous evaluations. Lancet.

[25] Baker, R. et al. Tailored interventions to address determinants of practice. *Cochrane Database Syst. Rev.* CD005470 (2015). 10.1002/14651858.CD005470.pub3 PMC7271646 25923419

[26] Flottorp SA (2013). A checklist for identifying determinants of practice: a systematic review and synthesis of frameworks and taxonomies of factors that prevent or enable improvements in healthcare professional practice. Implement. Sci..

[27] Carlsen B, Glenton C, Pope C (2007). Thou shalt versus thou shalt not: a meta-synthesis of GPs' attitudes to clinical practice guidelines. Br. J. Gen. Pract..

[28] Elward K, Blackburn B, Peterson LE, Greenawald M, Hagen MD (2014). Improving quality of care and guideline adherence for asthma through a group self-assessment module. J. Am. Board Fam. Med..

[29] Wisnivesky JP (2008). Barriers to adherence to asthma management guidelines among inner-city primary care providers. Ann. Allergy Asthma Immunol..

[30] Lugtenberg M, Zegers-van Schaick JM, Westert GP, Burgers JS (2009). Why don't physicians adhere to guideline recommendations in practice? An analysis of barriers among Dutch general practitioners. Implement. Sci..

[31] Navaratnam P, Jayawant SS, Pedersen CA, Balkrishnan R (2008). Asthma pharmacotherapy prescribing in the ambulatory population of the United States: evidence of nonadherence to national guidelines and implications for elderly people. J. Am. Geriatr. Soc..

[32] Scribano PV, Lerer T, Kennedy D, Cloutier MM (2001). Provider adherence to a clinical practice guideline for acute asthma in a pediatric emergency department. Acad. Emerg. Med..

[33] Chamberlain JM, Teach SJ, Hayes KL, Badolato G, Goyal MK (2016). Practice pattern variation in the care of children with acute asthma. Acad. Emerg. Med..

[34] O'Boyle CA, Henly SJ, Larson E (2001). Understanding adherence to hand hygiene recommendations: the theory of planned behavior. Am. J. Infect. Control.

[35] Jenner EA (2006). Discrepancy between self-reported and observed hand hygiene behaviour in healthcare professionals. J. Hosp. Infect..

[36] van Dulmen S (2011). The value of tailored communication for person-centred outcomes. J. Eval. Clin. Pract..

[37] van Hulten R, Blom L, Mattheusens J, Wolters M, Bouvy M (2011). Communication with patients who are dispensed a first prescription of chronic medication in the community pharmacy. Patient Educ. Couns..

[38] Driesenaar JA, De Smet PA, van Hulten R, Hu L, van Dulmen S (2016). Communication during counseling sessions about inhaled corticosteroids at the community pharmacy. Patient Prefer. Adherence.

[39] Driesenaar JA, De Smet PA, van Hulten R, Noordman J, van Dulmen S (2016). Cue-responding behaviors during pharmacy counseling sessions with patients with asthma about inhaled corticosteroids: potential relations with medication beliefs and self-reported adherence. Health Commun..

[40] van Mil JW, Schulz M, Tromp TF (2004). Pharmaceutical care, European developments in concepts, implementation, teaching, and research: a review. Pharm. world Sci..

[41] Horne R (2007). Can asthma control be improved by understanding the patient's perspective?. BMC Pulm. Med..

[42] Kooy MJ, van Geffen EC, Heerdink ER, van Dijk L, Bouvy ML (2014). Effects of a TELephone Counselling Intervention by Pharmacist (TelCIP) on medication adherence, patient beliefs and satisfaction with information for patients starting treatment: study protocol for a cluster randomized controlled trial. BMC Health Serv. Res..

[43] van Geffen EC, Kruijtbosch M, Egberts AC, Heerdink ER, van Hulten R (2009). Patients' perceptions of information received at the start of selective serotonin-reuptake inhibitor treatment: implications for community pharmacy. Ann. Pharmacother..

[44] Feifer RA, Greenberg L, Rosenberg-Brandl S, Franzblau-Isaac E (2010). Pharmacist counseling at the start of therapy: patient receptivity to offers of in-person and subsequent telephonic clinical support. Popul. Health Manag..

[45] SFK. SFK facts and figures on pharmaceutical care in the Netherlands in 2016 https://www.sfk.nl/english/facts-and-figures-2017 (2017).

[46] Koehler Tamara C., Bok Harold, Westerman Michiel, Jaarsma Debbie (2019). Developing a competency framework for pharmacy technicians: Perspectives from the field. Research in Social and Administrative Pharmacy.

[47] Buurma H (2008). Prevalence and determinants of pharmacy shopping behaviour. J. Clin. Pharm. Ther..

[48] Wet op de geneeskundige behandelingsovereenkomst (WGBO) http://wetten.overheid.nl/BWBR0005290/2016-04-01#Boek7_Titel7_Afdeling5 (2018).

[49] Teichert M, van der Aalst A, de Wit H, Stroo M, De Smet PA (2007). How useful are prescribing indicators based on the DU90% method to distinguish the quality of prescribing between pharmacotherapy audit meetings with different levels of functioning?. Eur. J. Clin. Pharmacol..

[50] Beyer M (2003). The development of quality circles/peer review groups as a method of quality improvement in Europe. Results of a survey in 26 European countries. Fam. Pract..

[51] Curtain C, Peterson GM (2014). Review of computerized clinical decision support in community pharmacy. J. Clin. Pharm. Ther..

